# Disparities in Internet Medical Service Utilization Among Patients in Post–COVID-19 China: Cross-Sectional Study of Data From Provincial Field and National Online Surveys

**DOI:** 10.2196/60546

**Published:** 2025-08-01

**Authors:** Zhenyu Sun, Xi Chen, Dongfu Qian

**Affiliations:** 1 School of Health Policy & Management, Nanjing Medical University, 101 Longmian Avenue, Nanjing, 211166, China, 86 15996273378; 2 School of Public Health, Yale University, New Haven, CT, United States; 3 Laboratory for Digital Intelligence & Health Governance, Nanjing Medical University, Nanjing, China

**Keywords:** internet medical services utilization, disparities, adult patients, status, patterns, preferences, COVID-19 pandemic, provincial field survey, national online survey, China

## Abstract

**Background:**

Internet medical services (IMS) expanded rapidly in China during the COVID-19 pandemic. Unfortunately, disparities in internet medical services utilization (IMSU) have marginalized disadvantaged groups of Chinese patients from digital health benefits. The extent and nature of these disparities remain poorly understood, with no research comprehensively addressing how unfavorable predictors, including demographic, socioeconomic, and health-related factors, shape IMSU status, patterns, and preferences in China after the COVID-19 pandemic.

**Objective:**

This study aims to gain a deeper understanding of the disparities and unfavorable predictors that limit IMSU among Chinese adult patients following the COVID-19 pandemic, providing key reference points for advancing equitable IMSU.

**Methods:**

This cross-sectional study used a triangular approach, combining data from a provincial field survey conducted in July 2023 and a national online survey conducted in March 2024. Participants were Chinese adult outpatients aged 18 years or older. Descriptive and comparative analyses were used to examine disparities in IMSU status, patterns, and preferences across different demographic, socioeconomic, and health status–related factors. Binary logistic regression models were applied to assess the associations between unfavorable predictors (constructed from selected demographic, socioeconomic, and health status–related factors) and IMSU status, patterns, and preferences.

**Results:**

Of the 2011 eligible participants in the Jiangsu provincial field survey, 787 (39.13%) reported using IMS at least once in the previous 12 months. Among the 1611 eligible participants in the national online survey, all reported accessing IMS during the same period; however, only 481 (29.86%) reported high-frequency use (defined as usage in the third quartile or above, ie, ≥6 times). Overall, participants with unfavorable predictors were less likely to engage in IMSU, deep IMS were used less frequently than shallow IMS, and participants with 6 or more cumulative unfavorable predictors had the lowest total of IMSU preference score (mean 48.98 and 57.37 in the provincial field and national online surveys, respectively). Based on combined data from the provincial field and national online surveys, significantly negative associations were observed between unfavorable predictors and IMSU status, patterns, and preferences. In particular, participants aged 60 years or above (odds ratio [OR] 0.40, 95% CI 0.25‐0.63, *P*<.001) and those without everyday internet access (OR 0.54, 95% CI 0.41‐0.71, *P*<.001) were the least likely to use IMS at a medium-to-high frequency. Participants without private health insurance (OR 0.59, 95% CI 0.44‐0.79, *P*<.001) were the least likely to utilize deep IMS. Moreover, participants aged 60 years or above (OR 0.45, 95% CI 0.33‐0.63, *P*<.001) and those with a high school education or less (OR 0.67, 95% CI 0.55‐0.82, *P*<.001) were the least likely to prefer IMS to a moderate-to-strong degree.

**Conclusions:**

Widespread disparities in IMSU status, patterns, and preferences persisted among Chinese adult patients after the COVID-19 pandemic. More pro-disadvantaged patient policies may be warranted to narrow these disparities in IMSU, such as reimbursement for IMSU, to promote digital health equity in China.

## Introduction

### Background

Internet medical services (IMS) refer to a range of health care–related services that are based on internet technologies and platforms, including health monitoring, online consultations and pharmacies, routine and follow-up visits, telemedicine, and so on [1-5]. IMS has been advancing steadily, and the COVID-19 pandemic further increased reliance on these services. For example, the percentage of US patients utilizing online medical records rose from 27.0% in 2014 and 28.2% in 2017 to 39.5% in 2020. Similarly, the cumulative number of visits to IMS-related platforms such as Ping An Good Doctor in China rapidly exceeded 1.1 billion between January and February in 2020 [6-10]. Nevertheless, as IMS become more central to health care, the inequalities in their utilization were also brought to light during the COVID-19 pandemic [11-14].

Despite evidence of patients’ continued interest in IMS and the sustained high levels of internet medical services utilization (IMSU) after the COVID-19 pandemic, equitable access remains a concern [15-19]. There are ongoing concerns that IMS may exacerbate disparities among patients who have limited access to the necessary infrastructure or lack the skills required for IMSU. These barriers tend to disproportionately impact older adults, rural residents, and individuals with disadvantaged socioeconomic backgrounds, such as lower levels of education, many of whom may already experience significant disparities in health behaviors and outcomes [20-22]. Theoretically, IMS offer the promise of improving access to medical services by removing cost barriers associated with management, information, traffic, and so on [23]. In practice, however, internet-based medical and other digital services risk widening health care gaps for individuals with inequitable sociodemographic characteristics (eg, living urban vs rural), rather than narrowing them [24,25].

### Study Objectives

If IMS continue to be a common and important approach to health care delivery in the postpandemic era, it is essential to systematically understand the disparities in the status, patterns, and preferences of IMSU for patients with inequalities in demographic, socioeconomic, and health-related factors, using multisource data. However, these disparities and the determinants of IMSU have not been well understood in China, particularly in the post–COVID-19 period. In this study, we adopted a triangular approach, combining data from a provincial field survey (conducted in July 2023) and a national online survey (conducted in March 2024), to assess IMSU among Chinese adult patients. Our aim was to identify disparities and unfavorable predictors in the status, patterns, and preferences of IMSU among patients with inequitable demographic, socioeconomic, and health-related characteristics. This analysis contributes to a deeper understanding of digital health inequalities in China and supports efforts to reduce them in the postpandemic context.

## Methods

### Settings and Participants

To mitigate the interference of common method biases on statistical results [26], and in light of our financial and human resource constraints, we used 2 survey methods, a provincial field survey and a national online survey, to enhance data reliability and the robustness of our conclusions. We used the Cochran formula for sample size estimation in cross-sectional studies as follows [27]:


$$N=\left[\frac{\mu_{\alpha }^{2}\pi (1-\pi )}{\delta^{2}}\right]$$

Generally, a 95% CI and a relative error of 0.02 are recommended. Thus, *μ^2^_α_*=1.96^2^=3.8416, and *δ^2^*=0.02^2^=0.0004 were used. According to the 51st Statistical Report on China’s Internet Development, as of December 2022, the number of IMS users had reached 363 million, accounting for 34% of the total internet users [28]. To obtain a conservative estimate of the theoretical sample size, we assumed an IMSU prevalence of 20%. Accordingly, the parameter *π*=0.2 (IMSU prevalence) was adopted. Substituting the above parameters into the Cochran formula, the theoretical sample size *N* was calculated to be 1537.

The provincial field survey was conducted in Jiangsu, a developed province located on China’s east coast, comprising 13 prefecture-level cities and a population of 85 million in 2022. A 3-stage stratified sampling was used to select participants. In the first stage, all prefecture-level cities in Jiangsu were stratified into 3 groups based on their economic and geographic characteristics in 2022. One city was then selected from each group. Accordingly, the cities of Changzhou (gross domestic product [GDP]: 950 billion Chinese yuan [CNY]; 1 CNY=US $0.14), Yangzhou (GDP: 711 billion CNY), and Lianyungang (GDP: 401 billion CNY) were chosen, representing southern, central, and northern Jiangsu, respectively. In the second stage, 1 district was selected from each sampled prefecture-level city based on annual GDP per capita. Accordingly, the districts of Wujin ( annual GDP per capita: 180,600 CNY) in Changzhou, Guangling (annual GDP per capita: 159,800 CNY) in Yangzhou, and Ganyu (annual GDP per capita: 72,700 CNY) in Lianyungang were selected. In the final stage, 3 hospitals (1 each at the primary, secondary, and tertiary levels) were chosen from each sampling district as the specific survey sites. The resident populations of Changzhou (5.37 million), Yangzhou (4.58 million), and Lianyungang (4.60 million) were similar in 2022. The distribution of primary, secondary, and tertiary hospitals in each prefecture-level city was regulated by the Jiangsu provincial government at an approximate ratio of 3:4:7. As a result, the survey was initially designed to enroll about 700 adult outpatients from each sampling prefecture-level city, with participant distribution across the selected primary, secondary, and tertiary hospitals also following the approximate 3:4:7 ratio, yielding a total of 2100 participants.

The national online survey was conducted via WJX, an online questionnaire survey platform owned by Changsha Ranxing Information Technology Co., Ltd in China. WJX recruited participants who were willing to take part in the survey from its sample bank of 6.2 million real ID–registered members. The survey was self-administered and accessible at any time during the designated period. Each participant was allowed to complete the survey only once and could exit at any point. Upon completion, WJX awarded participants with a small cash incentive. Based on economic and geographic characteristics, China is commonly divided into 4 regions: Eastern, Central, Western, and Northeastern. As of the end of 2022, these regions accounted for 564 million (40%), 365 million (26%), 383 million (27%), and 99 million (7%) of the total population, respectively. Therefore, this survey was initially designed to recruit approximately 1600 adult participants who had outpatient visits (offline or online) within the past 3 months, with the sampling distribution across the 4 regions approximating the 40:26:27:7 ratio. Additionally, during the actual survey, WJX voluntarily expanded our required target sample size by 10% as a complimentary benefit.

### Data Collection

The provincial field survey data were collected in July 2023 through face-to-face interviews conducted at the sampling hospitals. The interviews were administered to outpatients using a structured questionnaire by trained undergraduate and master’s students from the School of Health Policy & Management at Nanjing Medical University. During the interviews, participants completed paper-based questionnaires with the assistance of the interviewers to ensure that the questions were clearly understood and the responses accurately recorded. The completed paper questionnaires were carefully double-checked each day through mutual verification by team members and random quality inspections conducted by supervisors. Following this, the data were entered into an electronic database using EpiData 3.1 software (The EpiData Association) for further organization and analysis. The exclusion criteria were as follows: (1) age under 18 years; (2) premature withdrawal from the survey; (3) critically ill or emergency patients; (4) cases selected for propensity scoring; and (5) questionnaires with more than 10% missing data.

The national online survey data were collected in March 2024 by WJX under our supervision. To ensure data quality, we required WJX to randomly select participants and restrict each network internet protocol address to a single questionnaire submission, minimizing the risk of malicious or duplicate responses. Upon completing the survey, each participant received a 7 CNY incentive from WJX to encourage thoughtful and accurate responses. In addition, we requested WJX to conduct 3 waves of real-time quality control surveys, each involving approximately 500-600 participants, conducted at 1-week intervals. The exclusion criteria were (1) age under 18 years; (2) premature withdrawal; (3) critically ill or emergency patients; (4) cases selected for propensity scoring; (5) missing data exceeding 10%; and (6) response time of less than 3 minutes, as determined by a pilot test.

Regarding propensity scoring, participants whose scores on reverse-phrased items (after same-trend recoding) in the IMSU preferences scale (see Multimedia Appendix 1) exceeded 2 SDs above or below the mean were excluded to minimize bias from inattentive or patterned responses. After applying all exclusion criteria, 2011 participants from the provincial field survey and 1611 participants from the national online survey were deemed eligible for analysis. According to the Cochran formula for sample size estimation, the sample sizes of eligible participants from both the provincial field and national online surveys were considered sufficient.

### Variables and Measures

#### Internet Medical Services Utilization

The variables describing IMSU were constructed to capture its status, patterns, and preferences. First, following prior research [2,29], we used the number of times patients accessed IMS in the previous 12 months to define IMSU status. In the provincial field survey, IMSU status was categorized into 2 binary groups: “unused” (0 times) and “used” (≥1 times). By contrast, all participants in the national online survey reported having used IMS at least once during the past 12 months. Therefore, based on the national online survey data, IMSU status was divided into 2 frequency-based categories: “low frequency” (first quartile or less, ≤3 times) and “high frequency” (third quartile or more, ≥6 times).

Second, according to the Health Information National Trends Survey (HINTS) Briefs by the National Cancer Institute (National Institutes of Health) [11], we described the patterns of IMSU over the past 12 months in terms of commonly used platforms, primary reasons for use, and main purposes. The common platforms were categorized as follows: “internet hospital” (online services provided by public physical hospitals), “private platform” (private for-profit medical service platforms, eg, Ali Health), and “search engine” (web applications that return links to relevant web pages based on keywords or phrases entered, eg, Baidu). The primary reasons for IMSU included the following disease categories: “acute” (eg, appendicitis), “allergic” (eg, urticaria), “chronic” (eg, hypertension), “mental” (eg, anxiety), “minor” (eg, fever), and “urogenital” (eg, prostatitis). A minor disease refers to a self-limiting condition that typically resolves without specialized medical treatment, such as fever or influenza. Although chronic and acute diseases can progress to severe stages and potentially become major conditions that threaten survival, our questionnaire provided clear definitions, and trained investigators assisted participants in distinguishing between disease types to ensure accurate responses. The main purposes of IMSU included “health monitoring,” “drug purchase,” “routine visit,” “subsequent visit,” “teleconsultation,” “telediagnosis,” and “telesurgery.” The “tele-” series of IMS (eg, telediagnosis) refers to services in which a subordinate hospital initiates a request to a superior hospital via digital technology when it is unable to meet the patients’ needs. These services represent more advanced forms of IMS in China.

Based on previous studies [30-32], IMSU preferences were measured using a 4-dimensional, 7-point Likert scale developed by our research group (scores ranging from 1=strongly disagree to 7=strongly agree; see Multimedia Appendix 1). The 4 dimensions were common platforms, main purposes, media forms, and relative prices. The common platforms dimension included 3 items focusing on “internet hospital,” “private platform,” and “search engine.” The main purposes dimension included 3 items focusing on “routine visit,” “drug purchase,” and “telesurgery.” The media forms dimension included 3 items focusing on “image & text,” “phone call,” and “FaceTime.” The relative prices dimension included 3 items focusing on “less than offline,” “similar to offline,” and “higher than offline.” The scale demonstrated good validity and reliability. The Kaiser-Meyer-Olkin value was 0.87, the Bartlett test of sphericity was significant (*P*<.001), and the lowest Cronbach α among the dimensions was 0.69 (see Multimedia Appendix 2).

#### Demographic Factors

Demographic factors were age (<60 years or ≥60 years), gender (male or female), habitat (urban or rural), surveyed cities (Changzhou, Yangzhou, or Lianyungang in the provincial field survey), and surveyed regions (eastern, central, western, or northeastern in the national online survey).

#### Socioeconomic Factors

Socioeconomic factors were annual disposable income (≥30,000 CNY or <30,000 CNY), education level (higher than high school or high school or less), internet access (everyday or not everyday), private health insurance (have or without), level of hospitals surveyed (tertiary or primary or secondary), and work institutions (office or nonoffice).

#### Health Status–Related Factors

Health status–related factors were the number of chronic diseases (1 or fewer or 2 or more), which comprised cardiovascular (eg, hypertension), cerebral (eg, stroke), digestive (eg, pancreatitis), musculoskeletal (eg, osteoporosis), respiratory (eg, asthma), tumorous (eg, tumors), and other conditions (eg, lupus). Self-reported health status was also included, categorized as healthy, sub-health, and unhealthy. In the questionnaire, the responses “unhealthy” and “very unhealthy” were combined into a single unhealthy category.

### Statistical Analysis

In our study, all statistical analyses were performed using the R statistical software package (version 4.4.2; R Foundation). A 2-sided *P* value <.05 was considered statistically significant. Categorical variables were described using numbers and proportions (%), while continuous variables were presented as means and SDs. The Pearson chi-square test was used to compare categorical variables, and the Kruskal-Wallis *H* test was used to compare continuous variables. We performed binary logistic regression models using the R package “multinom” to evaluate associations between unfavorable predictors and status, patterns, and preferences of IMSU.

For executing the binary logistic regression models, we applied the quartile method to categorize IMSU status into binary groups: “low” (first quartile or less) and “medium-to-high” (above the first quartile). IMSU preferences were similarly divided into “weak” (first quartile or less) and “moderate-strong” (above the first quartile). IMSU patterns were classified into binary categories, namely, “shallow” and “deep,” using the K-means clustering method. Based on previous research [14,16,33,34], the pool of unfavorable predictors included 10 selected variables: 2 demographic factors (age and habitat), 6 socioeconomic factors (annual disposable income, education level, internet access, private health insurance, level of hospitals surveyed, and work institutions), and 2 health status–related factors (number of chronic diseases and self-reported health). Additionally, to enhance the robustness of our results, we repeatedly performed binary logistic regression models using the combined dataset from both the provincial field and national online surveys.

Drawing on the approach used in a previous study [33], we further constructed the variable “cumulative unfavorable predictors” to illustrate intergroup disparities in IMSU preferences. Each of the 10 dichotomized unfavorable predictors was assigned a value of 1 (unfavorable) or 0 (favorable), and the values were summed to generate a cumulative score. In this process, sub-health and unhealthy were merged into a single category. A higher score indicated the presence of more unfavorable predictors. All of the methods described above contributed to enhancing the robustness of our results and conclusions.

### Ethics Considerations

This study was reviewed and approved by the Ethics Committee of Nanjing Medical University (IRB Approval No. [2023]139). All participants provided informed consent before participation. The original consent form included provisions for the secondary use of deidentified data for research purposes. Participant privacy and confidentiality were strictly maintained throughout the study. All data were anonymized before analysis, and no personally identifiable information was retained. Participants in the Jiangsu provincial field survey received a towel as a token of appreciation, while participants in the national online survey were given a monetary reward of 7 CNY via transfer.

## Results

### Selected Characteristics of the Sampling Patients

Table 1 presents detailed demographic, socioeconomic, and health status information for the 2011 participants in the provincial field survey and the 1611 participants in the national online survey. In the provincial field survey, 836 (41.57%) participants were male and 1165 (57.93%) were female; 1812 (90.10%) were under 60 years old; 1218 (60.57%) lived in urban areas; 1362 (67.73%) had an annual disposable income of 30,000 CNY or above; 1047 (52.06%) had an education level higher than high school; 1545 (76.83%) reported everyday internet access; 90 (4.48%) had private health insurance; 1011 (50.27%) visited tertiary hospitals; 854 (42.47%) worked in office-based jobs; 1564 (77.77%) had 1 or fewer chronic diseases; and 897 (44.60%) reported being in good health. In the national online survey, 808 (50.16%) participants were male and 803 (49.84%) were female; 1584 (98.32%) were under 60 years old; 1531 (95.03%) lived in urban areas; 1538 (95.47%) had an annual disposable income of 30,000 CNY or above; 1499 (93.05%) had an education level higher than high school; 1351 (83.86%) reported everyday internet access; 351 (21.79%) had private health insurance; 927 (57.54%) visited tertiary hospitals; 1463 (90.81%) worked in office-based jobs; 850 (52.76%) had 1 or fewer chronic diseases; and 122 (7.57%) reported being in health.

**Table 1.** Selected characteristics of the participants.

Characteristics	Provincial field survey^a^, n (%)			National online survey^b^, n (%)			
	Changzhou (n=674)	Yangzhou (n=675)	Lianyungang (n=662)	Eastern (n=646)	Central (n=417)	Western (n=404)	Northeastern (n=144)
Demographic factors							
Age (years)							
<60	617 (91.5)	558 (82.7)	637 (96.2)	630 (97.5)	413 (99.0)	399 (98.8)	142 (98.6)
≥60	57 (8.5)	117 (17.3)	24 (3.6)	16 (2.5)	4 (1.0)	5 (1.2)	2 (1.4)
Gender							
Male	280 (41.5)	267 (39.6)	289 (43.7)	337 (52.2)	213 (51.1)	193 (47.8)	65 (45.1)
Female	389 (57.7)	407 (60.3)	369 (55.7)	309 (47.8)	204 (48.9)	211 (52.2)	79 (54.9)
Habitat							
Urban area	375 (55.6)	465 (68.9)	378 (57.1)	618 (95.7)	396 (95.0)	380 (94.1)	137 (95.1)
Rural area	297 (44.1)	209 (31.0)	279 (42.1)	28 (4.3)	21 (5.0)	24 (5.9)	7 (4.9)
Socioeconomic factors							
Annual disposable income (CNY^c^)							
≥30,000	457 (67.8)	444 (65.8)	461 (69.6)	620 (96.0)	400 (95.9)	381 (94.3)	137 (95.1)
<30,000	215 (31.9)	230 (34.1)	198 (29.9)	26 (4.0)	17 (4.1)	23 (5.7)	7 (4.9)
Education level							
Higher than high school	384 (57.0)	346 (51.3)	317 (47.9)	598 (92.6)	395 (94.7)	369 (91.3)	137 (95.1)
High school or less	290 (43.0)	329 (48.7)	342 (51.7)	48 (7.4)	22 (5.3)	35 (8.7)	7 (4.9)
Internet access							
Everyday	524 (77.7)	529 (78.4)	492 (74.3)	550 (85.1)	349 (83.7)	331 (81.9)	121 (84.0)
Not everyday	149 (22.1)	146 (21.6)	168 (25.4)	96 (14.9)	68 (16.3)	73 (18.1)	23 (16.0)
Private health insurance							
Have	27 (4.0)	43 (6.4)	20 (3.0)	141 (21.8)	97 (23.3)	85 (21.0)	28 (19.4)
Without	647 (96.0)	632 (93.6)	639 (96.5)	505 (78.2)	320 (76.7)	319 (79.0)	116 (80.6)
Level of hospitals surveyed							
Tertiary	338 (50.1)	341 (50.5)	332 (50.2)	376 (58.2)	247 (59.2)	220 (54.5)	84 (58.3)
Primary or secondary	336 (49.9)	334 (49.5)	330 (49.8)	270 (41.8)	170 (40.8)	184 (45.5)	60 (41.7)
Work institutions							
Office	308 (45.7)	289 (42.8)	257 (38.8)	608 (94.1)	375 (89.9)	347 (85.9)	133 (92.4)
Nonoffice	365 (54.2)	385 (57.0)	402 (60.7)	38 (5.9)	42 (10.1)	57 (14.1)	11 (7.6)
Health status–related factors							
Number of chronic diseases							
1 or fewer	530 (78.6)	488 (72.3)	546 (82.5)	369 (57.1)	221 (53.0)	196 (48.5)	64 (44.4)
2 or more	144 (21.4)	187 (27.7)	116 (17.5)	277 (42.9)	196 (47.0)	208 (51.5)	80 (55.6)
Self-reported health							
Healthy	308 (45.7)	269 (39.9)	320 (48.3)	53 (8.2)	32 (7.7)	32 (7.9)	5 (3.5)
Sub-health	330 (49.0)	355 (52.6)	305 (46.1)	476 (73.7)	308 (73.9)	298 (73.8)	105 (72.9)
Unhealthy	36 (5.3)	51 (7.6)	34 (5.1)	117 (18.1)	77 (18.5)	74 (18.3)	34 (23.6)

a The table excludes the missing values, so the total number is not always 2011 in the provincial field survey.

b No missing values in the national online survey.

c 1 CNY=US $0.14.

### Disparities in the Status of Internet Medical Services Utilization

**Figure 1.**
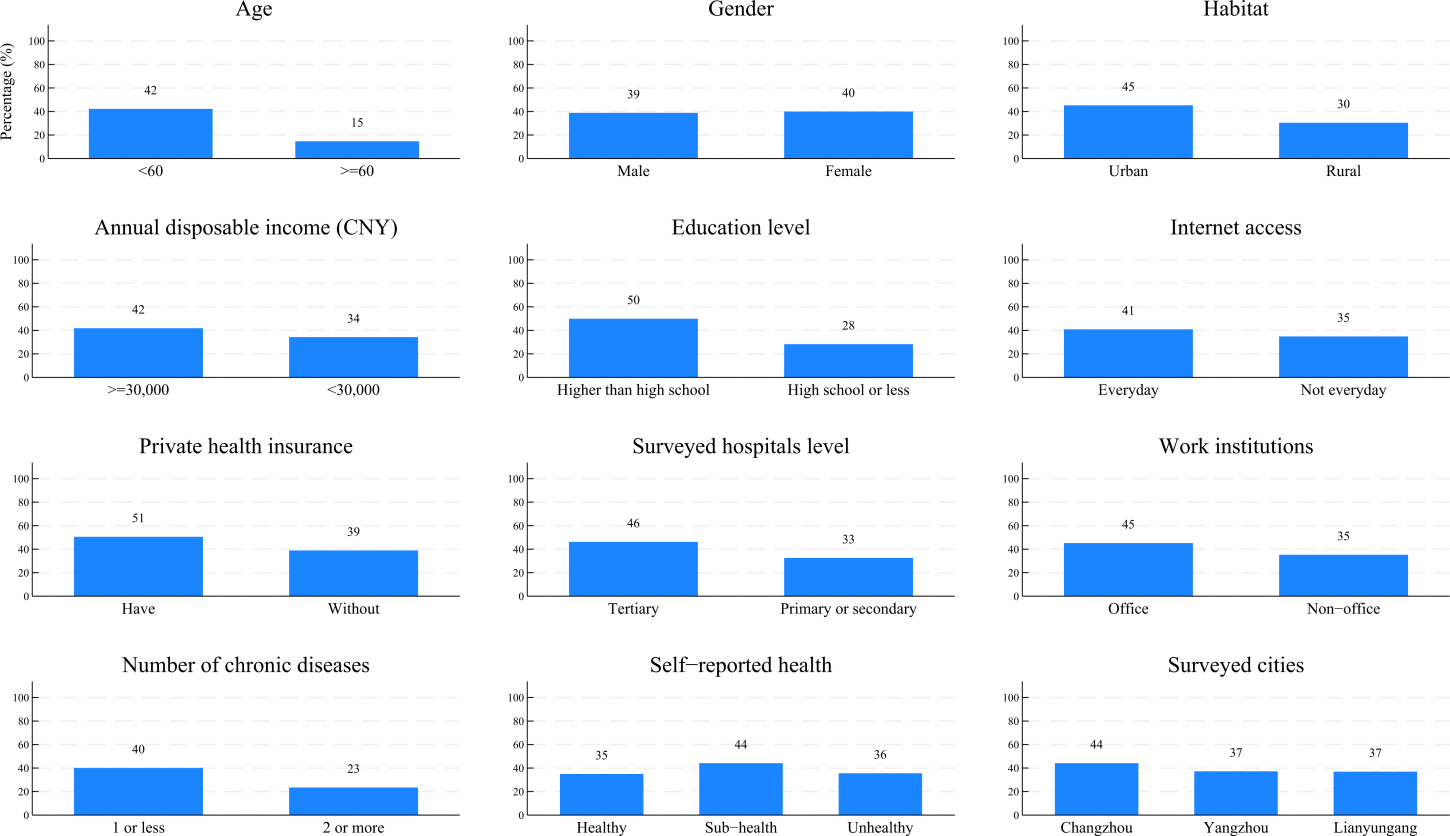
Disparities in internet medical services utilization status across demographic, socioeconomic, and health status–related factors, based on provincial field survey data. Blue bars represent the percentage of participants who reported accessing internet medical services in the past 12 months. 1 CNY=US $0.14.

Based on the provincial field survey data, Figure 1 and Table 2 present the disparities in IMSU status among participants with different demographic, socioeconomic, and health status–related characteristics. Overall, 787 (39.13%) participants in the provincial field survey conducted in July 2023 reported having accessed IMS during the past 12 months. Participants with disadvantaged demographic, socioeconomic, and health status–related characteristics were generally less exposed to IMSU (Figure 1).

**Table 2.** Association between internet medical services utilization status and unfavorable predictors based on binary logistic regression models.

Characteristics	Model 1^a^, odds ratio (95% CI)	Model 2^b^, odds ratio (95% CI)	Model 3^c^, odds ratio (95% CI)
Demographic factors			
Age (years)			
<60	Reference	Reference	Reference
≥60	0.41 (0.26-0.66)^d^	0.68 (0.31-1.52)	0.40 (0.25-0.63)^d^
Habitat			
Urban area	Reference	Reference	Reference
Rural area	0.79 (0.64-0.99)^e^	0.66 (0.14-3.04)	0.82 (0.66-1.00)^e^
Socioeconomic factors			
Annual disposable income (CNY^f^)			
≥30,000	Reference	Reference	Reference
<30,000	0.93 (0.73-1.18)	0.44 (0.26-0.75)^g^	0.83 (0.74-0.95)^e^
Education level			
Higher than high school	Reference	Reference	Reference
High school or less	0.59 (0.47-0.74)^d^	0.54 (0.36-0.83)^g^	0.58 (0.46-0.72)^d^
Internet access			
Everyday	Reference	Reference	Reference
Not everyday	0.54 (0.41-0.70)^d^	0.75 (0.56-1.00)^e^	0.54 (0.41-0.71)^d^
Private health insurance			
Have	Reference	Reference	Reference
Without	0.81 (0.52-1.26)	0.64 (0.49-0.85)^g^	0.82 (0.52-0.98)^e^
Level of surveyed hospitals			
Tertiary	Reference	Reference	Reference
Primary or secondary	0.78 (0.64-0.95)^e^	0.87 (0.70-1.08)	0.78 (0.64-0.95)^e^
Work institutions			
Office	Reference	Reference	Reference
Nonoffice	1.00 (0.80-1.26)	0.97 (0.69-1.53)	0.99 (0.79-1.24)
Health status–related factors			
Number of chronic diseases			
1 or fewer	Reference	Reference	Reference
2 or more	1.31 (1.00-1.72)^e^	1.77 (1.42-2.20)^d^	1.32 (1.01-1.73)^e^
Self-reported health			
Healthy	Reference	Reference	Reference
Sub-health	1.39 (1.14-1.71)^g^	1.10 (0.74-1.64)	1.39 (1.13-1.70)^g^
Unhealthy	1.29 (0.82-2.04)	2.11 (1.32-3.36)^g^	1.28 (0.81-2.02)

a Model 1 used data from the provincial field survey and was adjusted for surveyed cities, gender, internet medical services utilization patterns, and internet medical services utilization preferences.

b Model 2 used data from the national online survey and was adjusted for surveyed regions, gender, internet medical services utilization patterns, and internet medical services utilization preferences.

c Model 3 used combined data from both surveys and was adjusted for survey methods, gender, internet medical services utilization patterns, and internet medical services utilization preferences.

d *P*<.001.

e *P*<.05.

f 1 CNY=US $0.14.

g *P*<.01.

After adjusting for surveyed cities, gender, IMSU patterns, and IMSU preferences, provincial field survey participants aged 60 years or above (odds ratio [OR] 0.41, 95% CI 0.26‐0.66, *P*<.001), those living in rural areas (OR 0.79, 95% CI 0.64‐0.99, *P=*.04), with a high school education or less (OR 0.59, 95% CI 0.47‐0.74, *P*<.001), without everyday internet access (OR 0.54, 95% CI 0.41‐0.70, *P*<.001), and those visiting primary or secondary hospitals (OR 0.78, 95% CI 0.64‐0.95, *P*=.02) were significantly less likely to use IMS. By contrast, participants with 2 or more chronic diseases (OR 1.31, 95% CI 1.00‐1.72, *P*=.05), and those reporting sub-health (OR 1.39, 95% CI 1.14‐1.71, *P*=.00) were more likely to access IMS (model 1 in Table 2).

**Figure 2.**
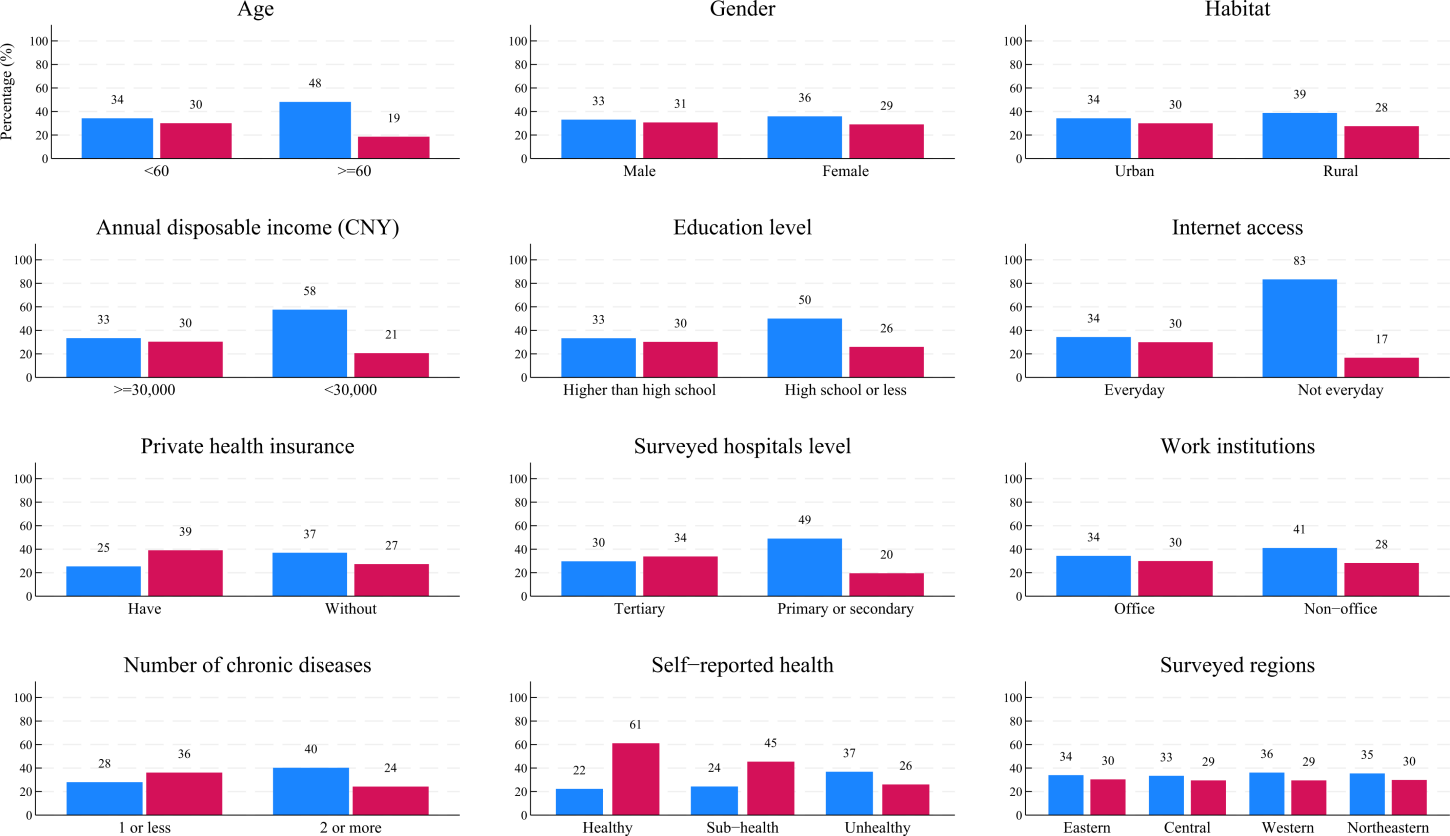
Disparities in internet medical services utilization status across demographic, socioeconomic, and health status–related factors, based on national online survey data. Blue bars represent the percentage of participants who reported low-frequency internet medical services use; red bars represent those who reported high-frequency internet medical services use in the past 12 months. 1 CNY=US $0.14.

Based on the national online survey data, Figure 2 and Table 2 show the disparities in IMSU status among participants with different demographic, socioeconomic, and health status–related characteristics. Overall, all participants (N=1611) in the national online survey conducted in March 2024 reported having accessed IMS during the past 12 months. Among them, 1056 (65.55%) accessed IMS at a low frequency (first quartile, ≤3 times), while 481 (29.86%) accessed it at a high frequency (third quartile, ≥6 times). Furthermore, participants with disadvantaged demographic, socioeconomic, and health status–related characteristics were more likely to access IMSU at a low frequency and less likely to access it at a high frequency (Figure 2).

After adjusting for surveyed regions, gender, IMSU patterns, and IMSU preferences, national online survey participants with an annual disposable income under 30,000 CNY (OR 0.44, 95% CI 0.26‐0.75, *P*<.001), high school education or less (OR 0.54, 95% CI 0.36‐0.83, *P*=.01), everyday internet access (OR 0.75, 95% CI 0.56‐1.00, *P*=.05), and no private health insurance (OR 0.64, 95% CI 0.49‐0.85, *P*<.001) were significantly less likely to use IMS at a medium-to-high frequency. By contrast, participants with 2 or more chronic diseases (OR 1.77, 95% CI 1.42‐2.20, *P*<.001) and those reporting being in an unhealthy state (OR 2.11, 95% CI 1.32‐3.36, *P*<.001) were significantly more likely to access IMS at a medium-to-high frequency (model 2 in Table 2).

For the mixed dataset, after adjusting for survey methods, gender, IMSU patterns, and IMSU preferences, participants from the provincial field and national online surveys who were aged 60 years or above (OR 0.40, 95% CI 0.25‐0.63, *P*<.001), lived in rural areas (OR 0.82, 95% CI 0.66‐1.00, *P=*.05), had an annual disposable income under 30,000 CNY (OR 0.83, 95% CI 0.74‐0.95, *P*=.01), had a high school education or less (OR 0.58, 95% CI 0.46‐0.72, *P*<.001), lacked everyday internet access (OR 0.54, 95% CI 0.41‐0.71, *P*<.001), had no private health insurance (OR 0.82, 95% CI 0.52‐0.98, *P*=.04), and visited primary or secondary hospitals (OR 0.78, 95% CI 0.64‐0.95, *P*=.01) were significantly less likely to use IMS at a medium-to-high frequency. By contrast, participants with 2 or more chronic diseases (OR 1.32, 95% CI 1.01‐1.73, *P*=.04) and those reporting sub-health (OR 1.39, 95% CI 1.13‐1.70, *P*<.001) were significantly more likely to access IMS at a medium-to-high frequency (model 3 in Table 2).

### Disparities in Patterns of Internet Medical Services Utilization

**Figure 3.**
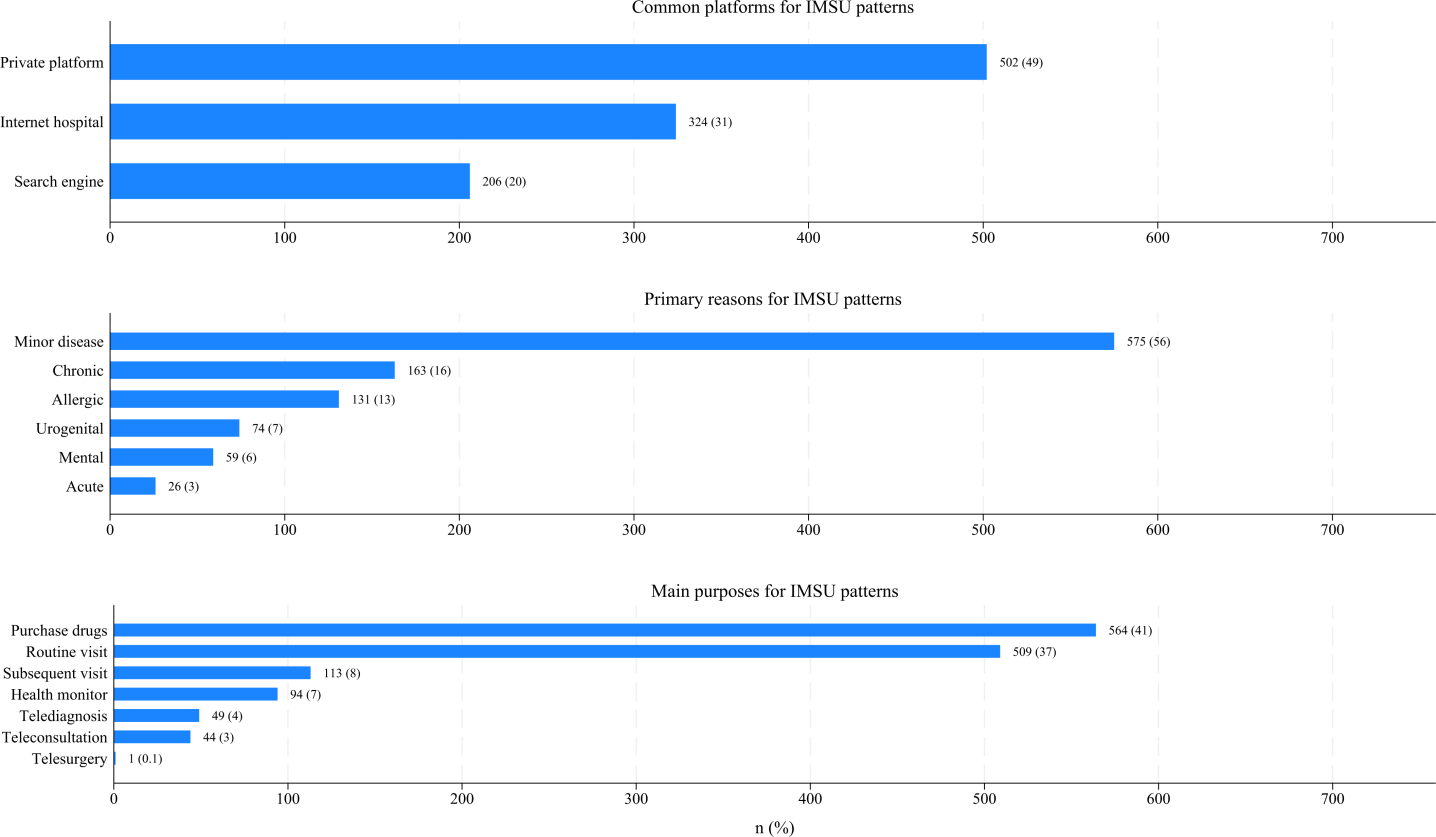
Patterns of internet medical services utilization (IMSU), including common platforms, primary reasons, and main purposes, based on provincial field survey data. Blue bars represent the frequency (percentage) of participants reporting each platform, reason, and purpose for using Internet medical services.

Based on the provincial field survey data, Figure 3 presents a snapshot of the patterns of IMSU. Among common platforms, private platforms were the most used (502/1032, percentage 48.64%), while search engines were the least used (206/1032, 19.96%). For primary reasons, minor diseases were the most frequently cited (575/1028, 55.93%), whereas acute diseases were the least common (26/1028, 2.52%). Regarding main purposes, drug purchase was the most prominent use case (564/1374, 41.04%), while telesurgery was the least used (1/1374, 0.07%).

After adjusting for surveyed cities, gender, IMSU status, and IMSU preferences, participants in the provincial field survey who lived in rural areas (OR 0.78, 95% CI 0.63‐0.97, *P=*.03), had a high school education or less (OR 0.73, 95% CI 0.58‐0.91, *P*=.01), lacked everyday internet access (OR 0.86, 95% CI 0.54‐0.97, *P=*.03), had no private health insurance (OR 0.94, 95% CI 0.60‐1.00, *P*=.05), or visited primary or secondary hospitals (OR 0.71, 95% CI 0.57‐0.95, *P*=.02) were significantly less likely to utilize deep IMS. By contrast, participants with 2 or more chronic diseases (OR 1.41, 95% CI 1.08‐1.85, *P*=.01) were significantly more likely to utilize deep IMS (model 1 in Table 3).

**Table 3.** Association between internet medical services utilization patterns and unfavorable predictors based on binary logistic regression models.

Characteristics	Model 1^a^, odds ratio (95% CI)	Model 2^b^, odds ratio (95% CI)	Model 3^c^, odds ratio (95% CI)
Demographic factors			
Age (years)			
<60	Reference	Reference	Reference
≥60	0.79 (0.54-1.15)	0.39 (0.17-0.89)^d^	0.73 (0.51-0.95)^d^
Habitat			
Urban area	Reference	Reference	Reference
Rural area	0.78 (0.63-0.97)^d^	0.76 (0.72-0.92)^d^	0.71 (0.56-0.91)^e^
Socioeconomic factors			
Annual disposable income (CNY)^f^			
≥30,000	Reference	Reference	Reference
<30,000	0.97 (0.82-1.30)	0.69 (0.40-0.79)^e^	0.97 (0.76-1.00)^d^
Education level			
Higher than high school	Reference	Reference	Reference
High school or less	0.73 (0.58-0.91)^e^	0.73 (0.46-0.86)^e^	0.82 (0.64-0.96)^d^
Internet access			
Everyday	Reference	Reference	Reference
Not everyday	0.86 (0.54-0.97)^d^	0.97 (0.71-1.33)	0.75 (0.57-0.98)^d^
Private health insurance			
Have	Reference	Reference	Reference
Without	0.94 (0.60-1.00)^d^	0.49 (0.36-0.67)^g^	0.59 (0.44-0.79)^g^
Level of hospitals surveyed			
Tertiary	Reference	Reference	Reference
Primary or secondary	0.71 (0.57-0.95)^d^	0.91 (0.80-1.28)	0.90 (0.67-0.94)^d^
Work institutions			
Office	Reference	Reference	Reference
Nonoffice	0.91 (0.72-1.14)	0.96 (0.63-1.47)	0.91 (0.72-1.17)
Health status–related factors			
Number of chronic diseases			
1 or fewer	Reference	Reference	Reference
2 or more	1.41 (1.08-1.85)^d^	3.47 (2.73-4.43)^g^	1.92 (1.58-2.32)^g^
Self-reported health			
Healthy	Reference	Reference	Reference
Sub-health	1.06 (0.87-1.30)	1.15 (0.54-1.35)	1.12 (0.90-1.39)
Unhealthy	1.20 (0.78-1.85)	1.21 (0.61-1.69)	1.06 (0.74-1.53)

a Model 1 used data from the provincial field survey and was adjusted for surveyed cities, gender, internet medical services utilization status, and internet medical services utilization preferences.

b Model 2 used data from the national online survey and was adjusted for surveyed regions, gender, internet medical services utilization status, and internet medical services utilization preferences.

c Model 3 used combined data from both surveys and was adjusted for survey methods, gender, internet medical services utilization status, and internet medical services utilization preferences.

d *P*<.05.

e *P*<.01.

f 1 CNY=US $0.14.

g *P*<.001.

**Figure 4.**
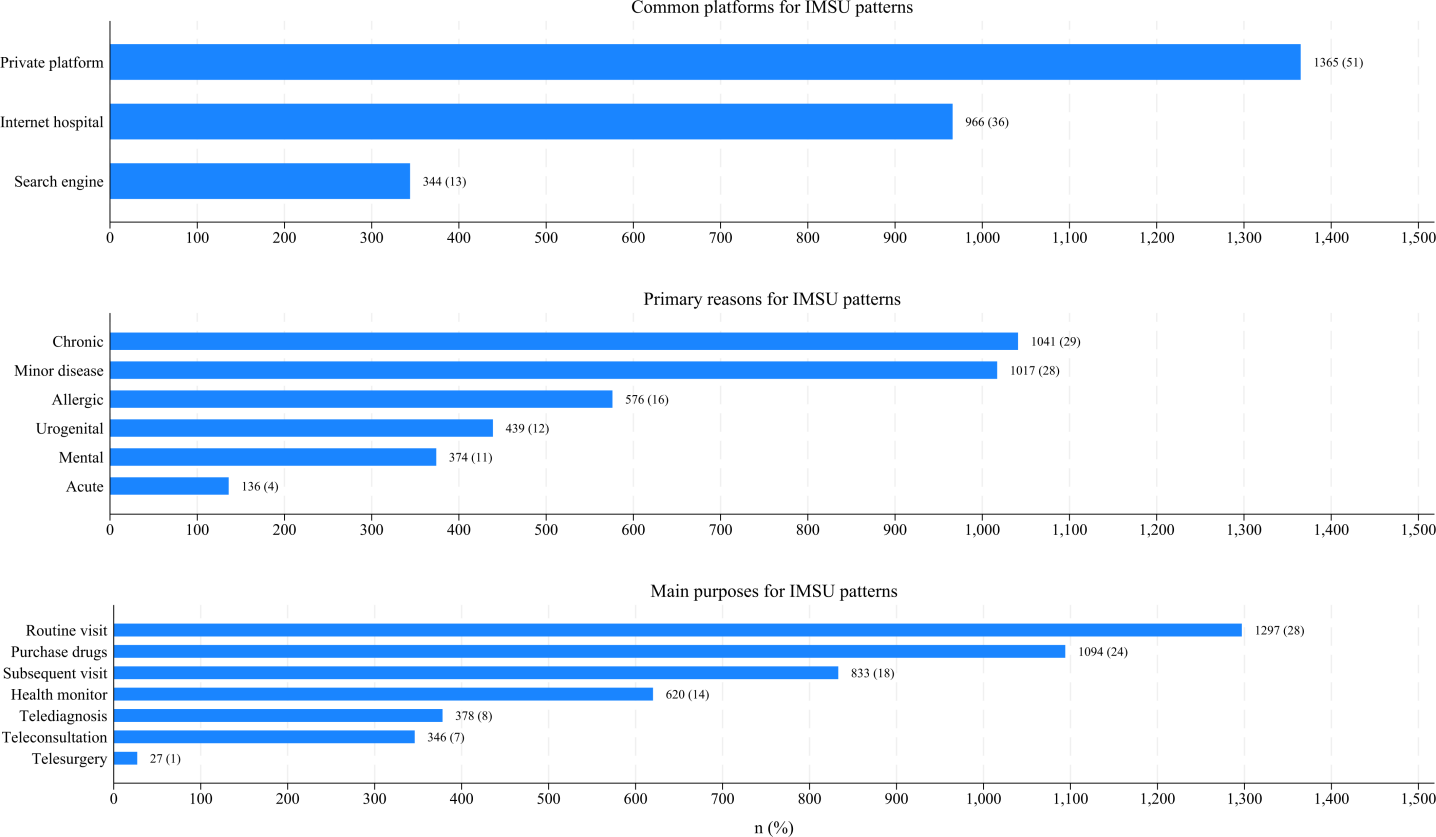
Patterns of internet medical services utilization (IMSU), including common platforms, primary reasons, and main purposes, based on national online survey data. Blue bars represent the frequency (percentage) of participants reporting each platform, reason, and purpose for utilizing internet medical services.

Based on the national online survey data, Figure 4 presents a snapshot of the patterns of IMSU. Among common platforms, private platforms were the most used (1365/2675, 51.02%), while search engines were the least used (344/2675, 12.85%). For primary reasons, chronic diseases were the most dominant (1041/3583, 29.05%), whereas acute diseases were the least common (136/3583, 3.79%). Regarding main purposes, routine visits were the most prominent (1297/4595, 28.22%), while telesurgery was the least utilized (27/4595, 0.58%).

After adjusting for surveyed regions, gender, IMSU status, and IMSU preferences, participants in the national online survey who were aged 60 years or above (OR 0.39, 95% CI 0.17‐0.89, *P=*.02), lived in rural areas (OR 0.76, 95% CI 0.72‐0.92, *P=*.01), had a high school education or less (OR 0.73, 95% CI 0.46‐0.86, *P*<.001), and had no private health insurance (OR 0.49, 95% CI 0.36‐0.67, *P*<.001) were significantly less likely to utilize deep IMS. By contrast, participants with 2 or more chronic diseases (OR 3.47, 95% CI 2.73‐4.43, *P*<.001) were significantly more likely to utilize deep IMS (model 2 in Table 3).

For the mixed dataset, after adjusting for survey methods, gender, IMSU status, and IMSU preferences, participants aged 60 years or above (OR 0.73, 95% CI 0.51‐0.95, *P=*.05), those living in rural areas (OR 0.71, 95% CI 0.56‐0.91, *P=*.01), with an annual disposable income under 30,000 CNY (OR 0.97, 95% CI 0.76‐1.00, *P*=.05), a high school education or less (OR 0.82, 95% CI 0.64‐0.96, *P*=.02), no everyday internet access (OR 0.75, 95% CI 0.57‐0.98, *P=*.03), no private health insurance (OR 0.59, 95% CI 0.44‐0.79, *P*<.001), and those who visited primary or secondary hospitals (OR 0.90, 95% CI 0.67‐0.94, *P*=.02) were significantly less likely to utilize deep IMS. Meanwhile, participants with 2 or more chronic diseases (OR 1.92, 95% CI 1.58‐2.32, *P*<.001) were significantly more likely to utilize deep IMS (model 3 in Table 3).

### Disparities in Preferences for Internet Medical Services Utilization

**Figure 5.**
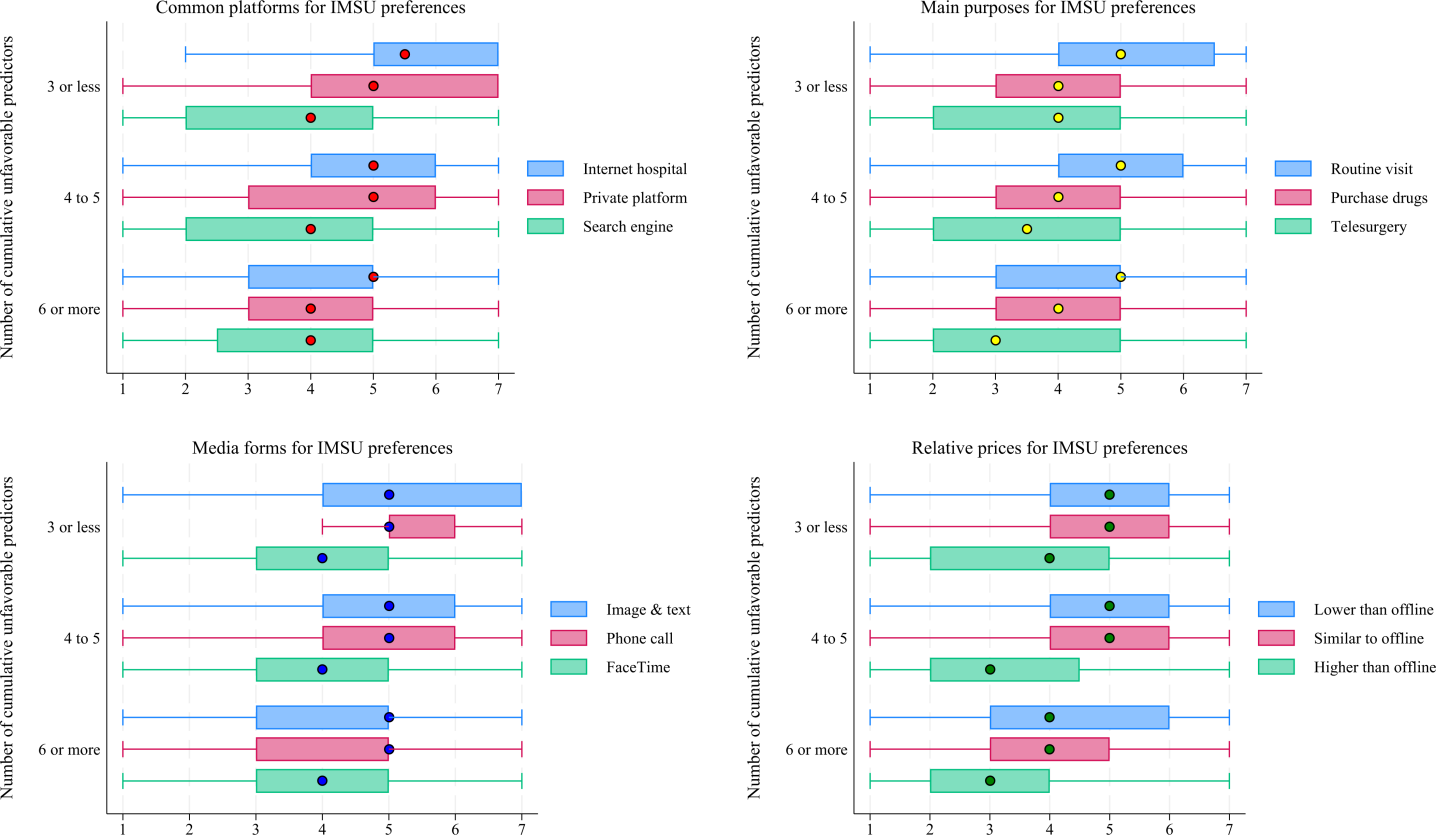
Disparities in internet medical services utilization (IMSU) preferences across cumulative unfavorable predictors, based on provincial field survey data. The number of cumulative unfavorable predictors was calculated by assigning a value of 0 to each favorable level and 1 to each unfavorable level, then summing 10 dichotomized predictors (with sub-health and unhealthy statuses merged). A higher total indicates the presence of more unfavorable predictors.

Based on the provincial field survey data, Figure 5 presents a panoramic view of the disparities in IMSU preferences among participants with different numbers of cumulative unfavorable predictors, comprising demographic, socioeconomic, and health status–related factors. Overall, participants with 3 or fewer cumulative unfavorable predictors had the highest total IMSU preference score (mean 56.39, SD 11.38), while those with 6 or more had the lowest total score (mean 48.98, SD 14.60). This disparity was statistically significant (*H*=71.44, *P*<.001). Detailed scores for IMSU preference dimensions—common platforms, main purposes, media forms, and relative prices—are shown in Figure 5.

After adjusting for surveyed cities, gender, IMSU status, and IMSU patterns, provincial field survey participants who were aged 60 years or above (OR 0.54, 95% CI 0.37‐0.78, *P*<.001), had an annual disposable income under 30,000 CNY (OR 0.87, 95% CI 0.68‐0.97, *P*=.02), had a high school education or less (OR 0.50, 95% CI 0.39‐0.64, *P*<.001), lacked everyday internet access (OR 0.72, 95% CI 0.55‐0.93, *P=*.01), and visited in primary or secondary hospitals (OR 0.70, 95% CI 0.55‐0.88, *P*<.001) were significantly less likely to prefer IMS at a moderate-to-strong degree (model 1 in Table 4).

**Table 4.** Association between internet medical services utilization preferences and unfavorable predictors based on binary logistic regression models.

Characteristics	Model 1^a^, odds ratio (95% CI)	Model 2^b^, odds ratio (95% CI)	Model 3^c^, odds ratio (95% CI)
Demographic factors			
Age (years)			
<60	Reference	Reference	Reference
≥60	0.54 (0.37-0.78)^d^	0.38 (0.17-0.85)^e^	0.45 (0.33-0.63)^f^
Habitat			
Urban area	Reference	Reference	Reference
Rural area	0.96 (0.81-1.32)	0.84 (0.51-1.39)	0.95 (0.86-1.29)
Socioeconomic factors			
Annual disposable income (CNY^g^)			
≥30,000	Reference	Reference	Reference
<30,000	0.87 (0.68-0.97)^e^	0.44 (0.26-0.76)^d^	0.78 (0.63-0.97)^e^
Education level			
Higher than high school	Reference	Reference	Reference
High school or less	0.50 (0.39-0.64)^f^	0.76 (0.49-0.95)^e^	0.67 (0.55-0.82)^f^
Internet access			
Everyday	Reference	Reference	Reference
Not everyday	0.72 (0.55-0.93)^e^	0.65 (0.48-0.87)^d^	0.74 (0.60-0.90)^d^
Private health insurance			
Have	Reference	Reference	Reference
Without	0.87 (0.76-2.13)	0.70 (0.56-0.88)^d^	0.74 (0.55-1.00)^e^
Level of surveyed hospitals			
Tertiary	Reference	Reference	Reference
Primary or secondary	0.70 (0.55-0.88)^d^	0.75 (0.60-0.94)^e^	0.79 (0.67-0.93)^d^
Work institutions			
Office	Reference	Reference	Reference
Nonoffice	1.00 (0.85-1.43)	0.76 (0.51-1.13)	0.97 (0.92-1.41)
Health status–related factors			
Number of chronic diseases			
1 or fewer	Reference	Reference	Reference
2 or more	0.98 (0.65-1.15)	1.12 (0.89-1.42)	1.00 (0.78-1.15)
Self-reported health			
Healthy	Reference	Reference	Reference
Sub-health	1.00 (0.79-1.26)	2.10 (1.41-3.11)^f^	1.05 (0.77-1.12)
Unhealthy	1.03 (0.46, 1.15)	2.60 (1.64-4.14)^f^	1.07 (0.81-1.41)

a Model 1 used data from the provincial field survey and was adjusted for surveyed cities, gender, internet medical services utilization status, and internet medical services utilization patterns.

b Model 2 used data from the national online survey and was adjusted for surveyed regions, gender, internet medical services utilization status, and internet medical services utilization patterns.

c Model 3 used combined data from both surveys and was adjusted for survey methods, gender, internet medical services utilization status, and internet medical services utilization patterns.

d *P*<.01.

e *P*<.05.

f *P*<.001.

g 1 CNY=US $0.14.

**Figure 6.**
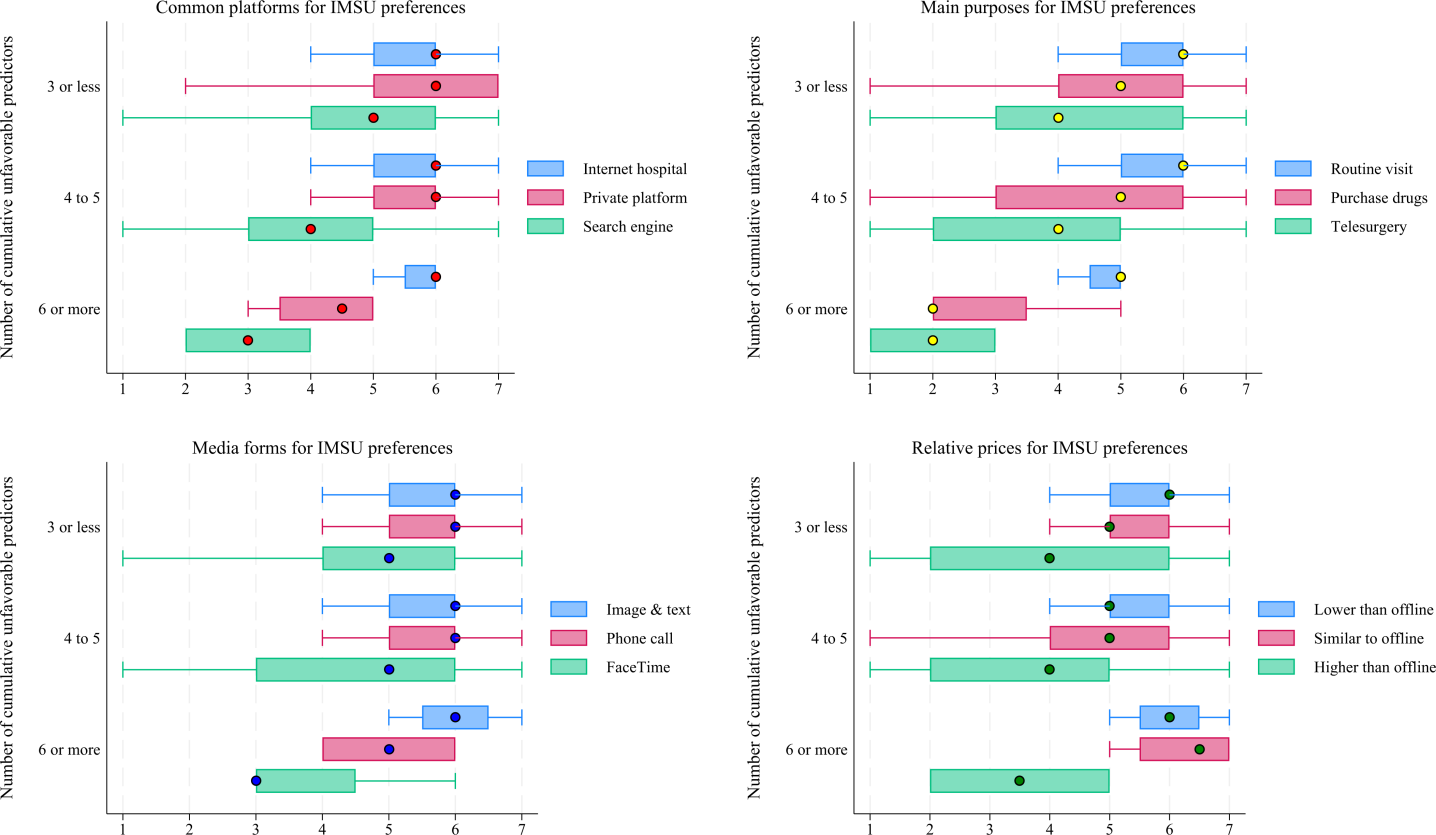
Disparities in internet medical services utilization (IMSU) preferences across cumulative unfavorable predictors, based on national online survey data. The number of cumulative unfavorable predictors was calculated by assigning a value of 0 to each favorable level and 1 to each unfavorable level, then summing 10 dichotomized predictors (with sub-health and unhealthy statuses merged). A higher total indicates the presence of more unfavorable predictors.

Based on the national online survey data, Figure 6 presents a panoramic view of the disparities in IMSU preferences among participants with different numbers of cumulative unfavorable predictors, consisting of demographic, socioeconomic, and health status–related factors. Overall, participants with 3 or fewer cumulative unfavorable predictors had the highest total IMSU preference score (mean 62.94, SD 9.95), while those with 6 or more had the lowest score (mean 57.37, SD 9.25). This disparity was statistically significant (*H*=29.94, *P*<.001). Detailed scores for the IMSU preference dimensions—common platforms, main purposes, media forms, and relative prices—are shown in Figure 6.

After adjusting for surveyed regions, gender, IMSU status, and IMSU patterns, national online survey participants who were aged 60 years or above (OR 0.38, 95% CI 0.17‐0.85, *P=*.02), had an annual disposable income under 30,000 CNY (OR 0.44, 95% CI 0.26‐0.76, *P*<.001), had a high school education or less (OR 0.76, 95% CI 0.49‐0.95, *P=*.02), lacked everyday internet access (OR 0.65, 95% CI 0.48‐0.87, *P*<.001), had no private health insurance (OR 0.70, 95% CI 0.56‐0.88, *P*<.001), and visited primary or secondary hospitals (OR 0.75, 95% CI 0.60‐0.94, *P*=.01) were significantly less likely to prefer IMS at a moderate-to-strong degree. By contrast, participants who reported sub-health (OR 2.10, 95% CI 1.41-3.11, *P*<.001) or unhealth (OR 2.60, 95% CI 1.64-4.14, *P*<.001) were significantly more likely to prefer IMS at a moderate-to-strong degree (model 2 in Table 4).

For the mixed data, after adjusting for survey methods, gender, IMSU status, and IMSU patterns, provincial field and national online survey participants who were aged 60 years or above (OR 0.45, 95% CI 0.33‐0.63, *P*<.001), had an annual disposable income under 30,000 CNY (OR 0.78, 95% CI 0.63‐0.97, *P*=.03), had high school or less education (OR 0.67, 95% CI 0.55‐0.82, *P*<.001), lacked everyday internet access (OR 0.74, 95% CI 0.60‐0.90, *P*<.001), had no private health insurance (OR 0.74, 95% CI 0.55‐1.00, *P=*.05), and visited primary or secondary hospitals (OR 0.79, 95% CI 0.67‐0.93, *P*<.001) were less likely to prefer IMS at a moderate-strong degree with statistical significance (model 3 in Table 4).

## Discussion

### Principal Findings and Implications

Based on a triangular approach combining a provincial field survey and a national online survey, this study identified disparities in the status, patterns, and preferences of IMSU among adult patients with unequal demographic, socioeconomic, and health status–related factors during the post–COVID-19 pandemic period in China. Several salient disparities were observed. First, regarding IMSU status, IMS were used less frequently among disadvantaged patient groups, and patients with unfavorable predictors were less likely to access IMS at a medium-to-high frequency. Second, in terms of IMSU patterns, deep IMS were used less often, and patients with unfavorable predictors were less likely to utilize deep IMS. Third, concerning IMSU preferences, patients with more unfavorable predictors were less likely to prefer IMS, tending instead to prefer shallow and lower-priced IMS. These findings suggest that disadvantaged patients remained at a disadvantage in IMSU in China after the COVID-19 pandemic.

The prevalence of IMSU we reported was similar to the latest data from the HINTS in the United States [11]. The characteristics of low IMSU rates we observed were consistent with prior studies on digital health inequities among geriatric, rural, and lower-educated patients, as well as the increased rate of IMSU among patients with multiple chronic diseases, though not with gender in this study [11,19,24,35-39]. Moreover, compared with patients from the provincial field survey, patients from the national online survey had superior demographic and socioeconomic characteristics, but poorer health status–related characteristics. Accordingly, patients from the national online survey accessed IMS more frequently and were more likely to increase the frequency, depth, and preference of IMSU based on their health status. These differential results may reflect the contribution of widespread digital device use and higher digital literacy in maximizing IMSU [40-42].

Previous studies have demonstrated that low-speed internet, lower annual income, no internet use, no health insurance, noncommercial health insurance, rural households, and older age were associated with a lower likelihood of engaging in deep IMS [39,40,43]. Our results significantly support these findings, showing that patients with disadvantaged demographic and socioeconomic factors were less likely to utilize deep IMS, and were mostly limited to shallow IMS, primarily provided by private platforms and internet hospitals. Prior research has also indicated that IMSU patterns tend to be deeper among patients with a higher number of chronic conditions and worse self-reported health status [11]. Regarding the number of chronic diseases, our results strongly support these findings. Nevertheless, for self-reported health, while our results align with prior arguments, the association was not statistically significant. This may be attributed to the inherent limitations of self-reporting in cross-sectional surveys [11,19,34,43-45].

Prior research has shown that patients with chronic diseases were widely interested in using telehealth, regardless of their sociodemographic factors, while geriatric patients tend to have a lower preference for IMS [10,30,46]. Our results are in line with these findings, but were not statistically significant. Further, our findings suggest that patients exposed to more unfavorable predictors tend to have a lower preference for IMSU and a higher preference for shallow and lower-priced IMS. In addition, patients’ IMSU preferences were partially mismatched with their actual IMSU patterns (eg, patients mostly preferred internet hospitals but used private platforms the most). The possible reasons may be related to patients’ online health usage habits and the supply of IMS across different platforms [6,32,41,47]. In addition, earlier studies have highlighted several patient-specific barriers, including the gap between preferences and actual behaviors, chronic disease conditions, limited digital health literacy, poor access to technology and the internet, and concerns over privacy and security, as well as policy-specific barriers, such as Medicare reimbursement for IMS [18,31,32,48,49].

More responsively, targeted strategies should be considered to address these disparities. For instance, policy measures that promote affordable internet access and smartphone ownership for disadvantaged patients, along with the integration of IMS into medical insurance reimbursement systems, could help reduce financial barriers [18,48]. Tailored digital literacy training and patient-centered IMS design can improve access and usability among patients who are older, have limited digital experience, or possess low levels of education and health literacy [40-42]. Additionally, integrating IMS with in-person health care models may help accommodate the preferences of disadvantaged patients who have lower digital trust [31,32]. Such strategies may be essential for promoting equitable access to IMS resources and reducing disparities in IMSU.

### Strengths and Limitations

A notable strength of this study is its position as the first cross-sectional study to employ a triangular approach, integrating a provincial field survey and a national online survey, to investigate disparities in the status, patterns, and preferences of IMSU among adult patients in China after the COVID-19 pandemic. Nonetheless, several limitations associated with cross-sectional data must be acknowledged. First, due to the inherent limitations of the field survey method, we were unable to assess the substitution effect of participants’ in-person visits on IMSU status, which may have resulted in an underestimation of IMSU usage. Second, the online survey method carries its own limitations, including a potentially low response rate among disadvantaged groups, particularly older patients and those with lower education, limited digital literacy, or restricted access to necessary digital infrastructure. Third, self-reported responses derived from the designated cross-sectional surveys may be subject to recall bias, and the cross-sectional design does not allow for causal inferences between unfavorable predictors and the status, patterns, and preferences of IMSU. Furthermore, the low participation of older adults (60 years and above) in both surveys, likely influenced by the survey distribution methods, such as field investigators favoring more cooperative middle-aged patients, and the higher proportion of younger registered members in the WJX sample bank, may have led to an underestimation of disparities within this subgroup. Accordingly, further extensive longitudinal and experimental studies are needed, including over-sampling of older adult participants, to better understand the disparities in IMSU status, patterns, and preferences.

Despite these limitations, we firmly believe that this study demonstrates the continued widespread unequal utilization of IMS between advantaged and disadvantaged groups of Chinese adult patients after the COVID-19 pandemic. Its contributions extend beyond China in at least two key areas. First, by using a triangular approach, combining a provincial field survey and a national online survey, we enhanced the prospective nature of the study and mitigated common method biases. As a result, our findings provide reliable insights into disparities in IMSU among adults with various unfavorable predictors. Second, in representative countries such as China that are actively advancing digitization, our results indicate that unequal IMS utilization may exacerbate health inequalities, contradicting the fundamental goal of using digitization to promote equitable access to health care. Accordingly, our study offers valuable implications from an IMSU perspective for both developed and developing countries facing digital health inequalities, including the need for medical insurance coverage for IMS, affordable internet and smartphone access, and individualized IMS systems tailored to disadvantaged patients.

### Conclusions

According to this cross-sectional study, disparities in IMSU remain prevalent among adult patients in China after the COVID-19 pandemic, with disadvantaged individuals consistently trailing in terms of IMSU status, patterns, and preferences. These findings underscore the need for pro-disadvantaged policies to advance IMS and promote digital health equity in China. In practice, greater efforts should focus on integrating IMS into medical insurance reimbursement, expanding internet access, and developing preference-based smart IMS referral systems tailored to low-income, older, and less-educated individuals, key reference points for Chinese health policy makers. Furthermore, a more comprehensive understanding and resolution of these disparities may require the integration of field, online, cross-sectional, longitudinal, and experimental studies. Such an approach can help ensure that disadvantaged patients are not left behind in the digital health transformation.

## Supplementary material

10.2196/60546 Multimedia Appendix 1 The English version of the questionnaire for the Internet Medical Services Utilization Survey.

10.2196/60546 Multimedia Appendix 2 The validity and reliability test for the Internet Medical Services Utilization Preferences Scale.

## Abbreviations

GDP: gross domestic product
HINTS: Health Information National Trends Survey
IMS: internet medical services
IMSU: internet medical services utilization
OR: odds ratio
