# Supplementary material for: Disparities in Internet Medical Service Utilization Among Patients in Post–COVID-19 China: Cross-Sectional Study of Data From Provincial Field and National Online Surveys
Source: J Med Internet Res. 2025 Aug 1;27:e60546. doi: 10.2196/60546 (PMC12316444; doi:10.2196/60546)
Supplement: Multimedia Appendix 1 [file jmir-v27-e60546-s001.docx]

**Multimedia Appendix 1**

**The English version of the questionnaire for internet medical services utilization survey.**

**Part 1: Basic Personal Information**

**1. Gender:**

🞎 Male

🞎 Female

**2. Age:**

___ years old

**3. Education Level:**

🞎 No formal education

🞎 Primary school

🞎 Junior high school

🞎 High school (or technical secondary school, vocational high school)

🞎 Junior college

🞎 Undergraduate

🞎 Postgraduate or above

**4. Work institutions** (if currently retired or unemployed, please select the last job held)**:**

🞎 Office-based (e.g., government/ public institution staff, company/enterprise employees)

🞎 Non-office-based (e.g., self-employed, freelancers, manual laborers, students, etc.)

**5. Annual Disposable Income:**

___ ×10,000 CNY

**6. Health Insurance Coverage (Multiple choices allowed):**

🞎 Public-funded medical care

🞎 Urban employee medical insurance

🞎 Urban and rural resident medical insurance

🞎 Private health insurance

🞎 No health insurance

**7. Habitat** (refers to the place where you have lived continuously for six months or longer)**:**

🞎 Urban

🞎 Rural

**8. Self-reported Health Status** (How you feel about your overall health)**:**

🞎 Health

🞎 Sub-health

🞎 Unhealth

🞎 Very unhealth

**9. Do You Have Any of the Following Chronic Diseases? (Multiple choices allowed):**

🞎 No chronic diseases

🞎 Cardiovascular (e.g., hypertension, diabetes)

🞎 Cerebral (e.g., infarction, stroke)

🞎 Respiratory (e.g., COPD, asthma)

🞎 Digestive system (e.g., pancreatitis, cholecystitis)

🞎 Musculoskeletal (e.g., rheumatism, osteoporosis)

🞎 Tumors

🞎 Others

**10. Net access (Frequency of Internet Use):**

🞎 Every day

🞎 Frequently week (at least 3-4 days a week)

🞎 Occasionally per week

🞎 Rarely or never use the internet

**11. In the Past 3 Months, the Online Doctor You Consulted Was From (If "Did not consult online" is selected, skip to Question 12; National Online Survey Item):**

🞎 Did not consult online

🞎 Primary hospital

🞎 Secondary hospital

🞎 Tertiary hospital

**Note:** In China, Primary hospitals are generally community or township-level hospitals. Secondary hospitals are usually county-level hospitals. Tertiary hospitals are generally municipal or higher-level hospitals.

**12. In the Past 3 Months, the Level of the Physical Hospital You Visited (Offline Visit) Was (National Online Survey Item):**

🞎 Primary hospital

🞎 Secondary hospital

🞎 Tertiary hospital

**Note:** In China, Primary hospitals are generally community or township-level hospitals. Secondary hospitals are usually county-level hospitals. Tertiary hospitals are generally municipal or higher-level hospitals.

**Part 2: Internet Medical Services Utilization**

**1. During the Past 12 Months, How Many Times Have You Used Internet Medical Services?**

___ times (If 0, skip to Question 6)

**2. During the Past 12 Months, Which Common Medical Service Platforms Have You Frequently Used? (Multiple choices allowed):**

🞎 Internet hospital (online operations of public physical hospitals)

🞎 Private platform (private for-profit medical services platforms, e.g., Ali Health, Haodf.com, Dingxiang Doctor, and Dingdang Quick Medicine, etc.)

🞎 Search engine (web applications that return links to relevant web pages through keywords or phrases entered, e.g., Baidu, QQ, and Sohu, etc.)

**3. During the Past 12 Months, What Were the Primary Reasons for Using Internet Medical Services? (Multiple choices allowed):**

🞎 Minor diseases (e.g., cold, fever)

🞎 Chronic diseases (e.g., hypertension, diabetes)

🞎 Acute diseases (e.g., appendicitis, acute heart failure)

🞎 Allergic diseases (e.g., asthma, urticaria)

🞎 Urogenital diseases (e.g., urology, gynecology)

🞎 Mental health issues (e.g., anxiety, depression)

🞎 Others___(Please specify)

**4. During the Past 12 Months, What Were the Main Purposes for Using Internet Medical Services? (Multiple choices allowed):**

🞎 Routine visit (using internet medical platforms for health and disease-related consultations, excluding free consultations)

🞎 Purchase drugs (Includes prescription drugs and over-the-counter (OTC) drugs)

🞎 Subsequent visit (using internet medical platforms for follow-up visits, re-examinations, and consistent drug prescriptions)

🞎 Home monitor (patients transmit real-time health and condition data to doctors via the internet, who then provide health improvement recommendations)

🞎 Teleconsultation (collaborative diagnosis and treatment planning between upper and lower-level hospitals via online platforms);

🞎 Telediagnosis (lower-level hospitals upload patient data, such as pathology, imaging, and ECG, to upper-level hospitals for diagnosis);

🞎 Telesurgery (upper-level hospitals perform surgeries on patients in lower-level hospitals via online platforms)

**6. The Internet Medical Services Utilization Preferences Scale**

| **Order** | **Items** | **Score** | | | | | | |
| --- | --- | --- | --- | --- | --- | --- | --- | --- |
|  |  | 1 | 2 | 3 | 4 | 5 | 6 | 7 |
| **Dimension 1: Common platforms** | | | | | | | | |
| 1 | I am willing to use the Internet hospital for diagnosis and treatment |  |  |  |  |  |  |  |
| 2 | I am willing to use the private platform for diagnosis and treatment |  |  |  |  |  |  |  |
| 3 | I am unwilling to use the search engine for diagnosis and treatment* |  |  |  |  |  |  |  |
| **Dimension 2: Main purposes** | | | | | | | | |
| 4 | I am willing to use Internet medical services for routine visit |  |  |  |  |  |  |  |
| 5 | I am unwilling to use Internet medical services for purchasing drugs* |  |  |  |  |  |  |  |
| 6 | I am unwilling to use Internet medical services for telesurgery* |  |  |  |  |  |  |  |
| **Dimension 3: Media forms** | | | | | | | | |
| 7 | I am willing to use image & text for accessing to Internet medical services |  |  |  |  |  |  |  |
| 8 | I am willing to use phone call for accessing to Internet medical services |  |  |  |  |  |  |  |
| 9 | I am unwilling to use FaceTime for accessing to Internet medical services* |  |  |  |  |  |  |  |
| **Dimension 4: Relative prices** | | | | | | | | |
| 10 | I am willing to use Internet medical services that are cheaper than offline medical services |  |  |  |  |  |  |  |
| 11 | I am willing to use Internet medical services with the similar price to offline medical services |  |  |  |  |  |  |  |
| 12 | I am unwilling to use Internet medical services that are more expensive than offline medical services* |  |  |  |  |  |  |  |

**Note:** 1=Strongly Disagree, 2=Disagree, 3=Slightly Disagree, 4=Neutral, 5=Slightly Agree, 6=Agree, and 7 =Strongly Agree. *indicated that the reverse-phrased item. Participants with a score of reverse-phrased items (after same-trend recoding) exceeding ±2 SDs from the mean were excluded, to minimize bias due to inattentive or patterned responses.

**Follow Information Completed by the Investigator (Just for the provincial field survey)**

**Survey Date:**

___ Year ___ Month ___ Day

**Survey Location:**

___ City ___ District/County

**Hospital Level:**

🞎 Primary hospital

🞎 Secondary hospital

🞎 Tertiary hospital

**Investigator's Signature: ___**
