# Supplementary material for: Disparities in Internet Medical Service Utilization Among Patients in Post–COVID-19 China: Cross-Sectional Study of Data From Provincial Field and National Online Surveys
Source: J Med Internet Res. 2025 Aug 1;27:e60546. doi: 10.2196/60546 (PMC12316444; doi:10.2196/60546)
Supplement: Multimedia Appendix 2 [file jmir-v27-e60546-s002.docx]

**Multimedia Appendix 2**

The validity and reliability test for the IMSU preferences scale

| **Dimensions** | **Cronbach's α values** | **KMO value** | **Bartlett test** |
| --- | --- | --- | --- |
| Common platforms | 0.82 | 0.87 | *P*<.001 |
| Main purposes | 0.69 |  |  |
| Media forms | 0.86 |  |  |
| Relative prices | 0.76 |  |  |
| Sample size | 3,622 | | |

Note: The data from the provincial field and national online surveys were mixed-used for analyzing the validity and reliability of the scale.
